# 4-Nitro­phenyl 4-bromo­benzoate

**DOI:** 10.1107/S1600536811043923

**Published:** 2011-10-29

**Authors:** Rodolfo Moreno-Fuquen

**Affiliations:** a Departamento de Química - Facultad de Ciencias, Universidad del Valle, Apartado 25360, Santiago de Cali, Colombia

## Abstract

In the crystal structure of the title compound, C_13_H_8_BrNO_4_, mol­ecules are linked into chains along [101] by weak C—H⋯O hydrogen bonds and Br⋯O contacts [3.140 (4) Å]. The planes of the nitrated and brominated aryl rings form a dihedral angle of 64.98 (10)°, indicating a twist in the mol­ecule.

## Related literature

For background to the applications of aromatic esters containing nitro groups, see: Jefford & Zaslona (1985 ▶). For mol­ecular and supra­molecular structures of nitroaryl compounds, see: Wardell *et al.* (2005 ▶); Jefford *et al.*, (1986 ▶). For halogen bonding, see: Politzer *et al.* (2010 ▶); Ritter (2009 ▶). For hydrogen bonding, see: Nardelli (1995 ▶) and for hydrogen-bond graph-set motifs, see: Etter (1990 ▶).
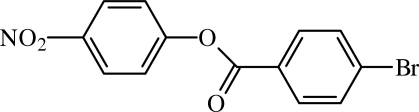


## Experimental

### 

#### Crystal data

C_13_H_8_BrNO_4_
*M* *_r_* = 322.11
Monoclinic, 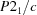

*a* = 8.8177 (4) Å
*b* = 9.5279 (5) Å
*c* = 14.9394 (5) Å
β = 99.024 (3)°
*V* = 1239.59 (10) Å^3^
*Z* = 4
Mo *K*α radiation
μ = 3.33 mm^−1^
*T* = 293 K
0.55 × 0.31 × 0.23 mm

#### Data collection

Bruker–Nonius KappaCCD diffractometer
Absorption correction: multi-scan (*SADABS*; Sheldrick, 1996 ▶) *T*_min_ = 0.250, *T*_max_ = 0.361
9341 measured reflections
2648 independent reflections
1918 reflections with *I* > 2σ(*I*)
*R*_int_ = 0.070

#### Refinement

*R*[*F* ^2^ > 2σ(*F* ^2^)] = 0.048
*wR*(*F* ^2^) = 0.137
*S* = 1.02
2648 reflections
172 parameters
H-atom parameters constrained
Δρ_max_ = 0.80 e Å^−3^
Δρ_min_ = −0.68 e Å^−3^

### 

Data collection: *COLLECT* (Nonius, 1998 ▶); cell refinement: *SCALEPACK* (Otwinowski & Minor, 1997 ▶); data reduction: *DENZO* (Otwinowski & Minor, 1997 ▶) and *SCALEPACK*; program(s) used to solve structure: *SHELXS97* (Sheldrick, 2008 ▶); program(s) used to refine structure: *SHELXL97* (Sheldrick, 2008 ▶); molecular graphics: *ORTEP-3 for Windows* (Farrugia, 1997 ▶) and *Mercury* (Macrae *et al.*, 2006 ▶); software used to prepare material for publication: *WinGX* (Farrugia, 1999 ▶).

## Supplementary Material

Crystal structure: contains datablock(s) I, global. DOI: 10.1107/S1600536811043923/hg5114sup1.cif

Structure factors: contains datablock(s) I. DOI: 10.1107/S1600536811043923/hg5114Isup2.hkl

Supplementary material file. DOI: 10.1107/S1600536811043923/hg5114Isup3.cml

Additional supplementary materials:  crystallographic information; 3D view; checkCIF report

## Figures and Tables

**Table 1:** Hydrogen-bond geometry (Å, °)

*D*—H⋯*A*	*D*—H	H⋯*A*	*D*⋯*A*	*D*—H⋯*A*
C10—H10⋯O4^i^	0.93	2.69	3.543 (6)	153
C3—H3⋯O3^ii^	0.93	2.60	3.335 (5)	136
C13—H13⋯O3^iii^	0.93	2.67	3.460 (5)	143
C12—H12⋯O1^iv^	0.93	2.50	3.237 (5)	137

Symmetry codes: (i) 
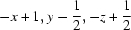
; (ii) 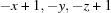; (iii) 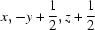; (iv) 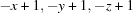.

**Fig. 1.**
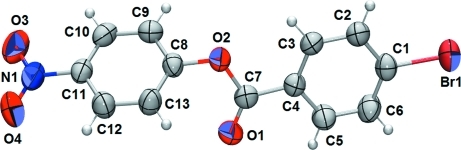
An ORTEP-3 (Farrugia, 1997) plot of (I) with displacement ellipsoids drawn at the 50% probability level. H atoms are shown as spheres of arbitrary radius.

**Fig. 2.**
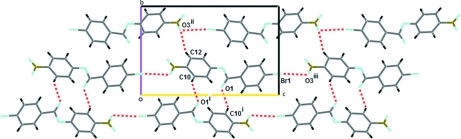
Part of the crystal structure of (I), showing the formation of a one dimensional sheet along [101]. Symmetry code: (i) -x,-y,-z + 1; (ii) -x,+y + 1/2,-z + 1/2; (iii) x - 1,+y,+z + 1.

## supplementary crystallographic information

### Comment

Aromatic esters containing nitro groups in their aromatic rings can be used as precursors for the preparation of compounds with potential analgesic and anti-inflammatory properties (Jefford & Zaslona, 1985). Molecular and supramolecular structures of a wide range of nitroaryl compounds have been reported (Wardell *et al.*, 2005 and Jefford *et al.*, 1986).

In order to complement the structural information on nitroaryl compounds the title ester, 4-nitrophenyl bromobenzoate (I) was synthesized. A perspective view of the molecule of the title compound, showing the atomic numbering scheme, is given in Fig. 1. The central ester fragment between atoms C4 and C8 is effectively planar. The nitrated and brominated aryl rings form a dihedral angle of 64.98 (10)°, indicating a twist in the molecule. The nitro group forms a dihedral angle of 2.7 (5)° with the adjacent aryl ring. Halogen bonding, an electrostatically driven higly directional noncovalent interaction, that can be important for its potential in the development of new materials and pharmaceutical compounds (Politzer *et al.*, 2010 and Ritter, 2009) can be observed in the present structure. Indeed, the Br···O contacts along [101] with a Br1···O3^iii^, (iii: *x* - 1,+*y*,+*z* + 1) distance of 3.140 (4) Å, showing the formation of an infinite chain is detected (see Fig. 2). Other C—H···O weak hydrogen bonds (see Table 1, Nardelli, 1995) that complement the crystal packing can also be seen in this figure. The propagation of these interactions forms *R*^3^_3_(30), *R*^4^_4_(24) and *R*^2^_2_(14) rings (Etter, 1990) along this direction.

### Experimental

Solution containing equimolar quantities (3.2 mmol) of 4-bromobenzoyl chloride and 4-nitrophenol in acetonitrile (60 ml) was gradually heated under reflux for 2 h. At room temperature, triethylamine was added, to get a solid which was poured in cold water. The solid was recrystallized in dichlorometane to yield excellent yellow crystals suitable for single-crystal X-ray diffraction. *M*.p. 431 (1) K.

### Refinement

The H-atoms were placed geometrically [C—H= 0.93 Å, *U*_iso_(H) (1.2 times *U*_eq_ of the parent atom].

### Crystal data

**Table:**

C_13_H_8_BrNO_4_	*F*(000) = 640
*M**_r_* = 322.11	*D*_x_ = 1.726 Mg m^−^^3^
Monoclinic, *P*2_1_/*c*	Melting point: 431(1) K
Hall symbol: -P 2ybc	Mo *K*α radiation, λ = 0.71073 Å
*a* = 8.8177 (4) Å	Cell parameters from 5487 reflections
*b* = 9.5279 (5) Å	θ = 2.9–27.1°
*c* = 14.9394 (5) Å	µ = 3.33 mm^−^^1^
β = 99.024 (3)°	*T* = 293 K
*V* = 1239.59 (10) Å^3^	Block, pale-yellow
*Z* = 4	0.55 × 0.31 × 0.23 mm

### Data collection

**Table:**

Bruker–Nonius KappaCCD diffractometer	2648 independent reflections
Radiation source: fine-focus sealed tube	1918 reflections with *I* > 2σ(*I*)
graphite	*R*_int_ = 0.070
ω scans	θ_max_ = 27.1°, θ_min_ = 3.5°
Absorption correction: multi-scan (*SADABS*; Sheldrick, 1996)	*h* = −10→11
*T*_min_ = 0.250, *T*_max_ = 0.361	*k* = −11→11
9341 measured reflections	*l* = −19→16

### Refinement

**Table:**

Refinement on *F*^2^	Primary atom site location: structure-invariant direct methods
Least-squares matrix: full	Secondary atom site location: difference Fourier map
*R*[*F*^2^ > 2σ(*F*^2^)] = 0.048	Hydrogen site location: inferred from neighbouring sites
*wR*(*F*^2^) = 0.137	H-atom parameters constrained
*S* = 1.02	*w* = 1/[σ^2^(*F*_o_^2^) + (0.0723*P*)^2^ + 0.6227*P*] where *P* = (*F*_o_^2^ + 2*F*_c_^2^)/3
2648 reflections	(Δ/σ)_max_ < 0.001
172 parameters	Δρ_max_ = 0.80 e Å^−^^3^
0 restraints	Δρ_min_ = −0.68 e Å^−^^3^

### Special details

**Table:**

Geometry. All e.s.d.'s (except the e.s.d. in the dihedral angle between two l.s. planes) are estimated using the full covariance matrix. The cell e.s.d.'s are taken into account individually in the estimation of e.s.d.'s in distances, angles and torsion angles; correlations between e.s.d.'s in cell parameters are only used when they are defined by crystal symmetry. An approximate (isotropic) treatment of cell e.s.d.'s is used for estimating e.s.d.'s involving l.s. planes.
Refinement. Refinement of *F*^2^ against ALL reflections. The weighted *R*-factor *wR* and goodness of fit *S* are based on *F*^2^, conventional *R*-factors *R* are based on *F*, with *F* set to zero for negative *F*^2^. The threshold expression of *F*^2^ > σ(*F*^2^) is used only for calculating *R*-factors(gt) *etc*. and is not relevant to the choice of reflections for refinement. *R*-factors based on *F*^2^ are statistically about twice as large as those based on *F*, and *R*- factors based on ALL data will be even larger.

### Fractional atomic coordinates and isotropic or equivalent isotropic displacement parameters (Å^2^)

**Table:**

	*x*	*y*	*z*	*U*_iso_*/*U*_eq_	
Br	1.07826 (4)	0.25892 (4)	1.01950 (2)	0.0659 (2)	
O2	0.6701 (3)	0.1952 (3)	0.59586 (16)	0.0569 (6)	
C1	0.9755 (4)	0.2726 (4)	0.8984 (2)	0.0507 (8)	
O1	0.7563 (3)	0.4159 (3)	0.58276 (16)	0.0606 (6)	
C4	0.8266 (4)	0.2946 (3)	0.7227 (2)	0.0457 (7)	
C8	0.5953 (4)	0.1968 (4)	0.5064 (2)	0.0480 (7)	
C11	0.4518 (4)	0.1846 (4)	0.3321 (2)	0.0504 (8)	
C10	0.5615 (4)	0.0861 (4)	0.3623 (2)	0.0552 (8)	
H10	0.5859	0.0161	0.3235	0.066*	
N1	0.3778 (5)	0.1834 (4)	0.2373 (2)	0.0694 (9)	
C7	0.7503 (4)	0.3138 (4)	0.6281 (2)	0.0485 (7)	
C5	0.9242 (4)	0.4006 (4)	0.7604 (3)	0.0594 (9)	
H5	0.9388	0.4796	0.7261	0.071*	
C2	0.8757 (4)	0.1668 (4)	0.8629 (2)	0.0527 (8)	
H2	0.8587	0.0893	0.8978	0.063*	
C3	0.8023 (4)	0.1793 (3)	0.7748 (2)	0.0499 (8)	
H3	0.7354	0.1090	0.7499	0.060*	
C6	0.9997 (5)	0.3897 (4)	0.8482 (2)	0.0631 (10)	
H6	1.0659	0.4603	0.8732	0.076*	
C9	0.6350 (4)	0.0922 (3)	0.4507 (2)	0.0535 (8)	
H9	0.7101	0.0268	0.4724	0.064*	
C13	0.4817 (4)	0.2937 (4)	0.4769 (2)	0.0554 (8)	
H13	0.4546	0.3616	0.5162	0.066*	
O3	0.4181 (5)	0.0922 (4)	0.18766 (19)	0.0955 (11)	
C12	0.4100 (5)	0.2880 (4)	0.3889 (3)	0.0580 (9)	
H12	0.3340	0.3527	0.3674	0.070*	
O4	0.2819 (6)	0.2721 (4)	0.2116 (3)	0.1064 (14)	

### Atomic displacement parameters (Å^2^)

**Table:**

	*U*^11^	*U*^22^	*U*^33^	*U*^12^	*U*^13^	*U*^23^
Br	0.0611 (3)	0.0868 (3)	0.0476 (3)	−0.00760 (19)	0.00100 (18)	−0.00065 (17)
O2	0.0682 (16)	0.0504 (12)	0.0479 (13)	−0.0114 (12)	−0.0034 (11)	0.0040 (11)
C1	0.0421 (17)	0.062 (2)	0.0475 (18)	0.0014 (14)	0.0051 (14)	−0.0012 (14)
O1	0.0684 (17)	0.0519 (14)	0.0599 (14)	−0.0047 (11)	0.0054 (12)	0.0080 (11)
C4	0.0445 (17)	0.0475 (16)	0.0456 (17)	0.0002 (14)	0.0079 (14)	−0.0012 (14)
C8	0.0480 (19)	0.0511 (17)	0.0434 (16)	−0.0075 (14)	0.0029 (14)	0.0044 (14)
C11	0.059 (2)	0.0515 (18)	0.0408 (16)	−0.0151 (16)	0.0091 (15)	0.0008 (14)
C10	0.067 (2)	0.0478 (18)	0.0534 (19)	−0.0115 (16)	0.0189 (17)	−0.0075 (14)
N1	0.094 (3)	0.067 (2)	0.0456 (17)	−0.030 (2)	0.0054 (17)	0.0047 (16)
C7	0.0460 (18)	0.0476 (18)	0.0531 (19)	−0.0014 (14)	0.0112 (15)	−0.0003 (15)
C5	0.060 (2)	0.058 (2)	0.059 (2)	−0.0160 (17)	0.0039 (16)	0.0070 (16)
C2	0.058 (2)	0.0476 (18)	0.0524 (18)	0.0002 (15)	0.0073 (16)	−0.0007 (14)
C3	0.054 (2)	0.0441 (17)	0.0509 (18)	−0.0044 (14)	0.0059 (15)	−0.0034 (14)
C6	0.061 (2)	0.067 (2)	0.059 (2)	−0.0197 (18)	0.0020 (18)	−0.0030 (17)
C9	0.057 (2)	0.0445 (17)	0.059 (2)	−0.0004 (15)	0.0105 (16)	0.0020 (14)
C13	0.056 (2)	0.0594 (19)	0.050 (2)	0.0043 (17)	0.0057 (16)	−0.0083 (16)
O3	0.148 (3)	0.092 (2)	0.0467 (15)	−0.027 (2)	0.0140 (18)	−0.0131 (15)
C12	0.057 (2)	0.062 (2)	0.053 (2)	0.0049 (17)	0.0034 (17)	0.0015 (16)
O4	0.137 (4)	0.105 (3)	0.063 (2)	0.011 (2)	−0.028 (2)	0.0082 (17)

### Geometric parameters (Å, °)

**Table:**

Br—C1	1.896 (4)	C10—C9	1.379 (5)
O2—C7	1.379 (4)	C10—H10	0.9300
O2—C8	1.394 (4)	N1—O4	1.214 (5)
C1—C6	1.380 (5)	N1—O3	1.230 (5)
C1—C2	1.387 (5)	C5—C6	1.379 (5)
O1—C7	1.192 (4)	C5—H5	0.9300
C4—C3	1.383 (5)	C2—C3	1.378 (5)
C4—C5	1.388 (5)	C2—H2	0.9300
C4—C7	1.477 (5)	C3—H3	0.9300
C8—C9	1.378 (5)	C6—H6	0.9300
C8—C13	1.383 (5)	C9—H9	0.9300
C11—C10	1.372 (5)	C13—C12	1.367 (5)
C11—C12	1.387 (5)	C13—H13	0.9300
C11—N1	1.465 (4)	C12—H12	0.9300
			
C7—O2—C8	117.8 (3)	C6—C5—C4	120.5 (3)
C6—C1—C2	121.5 (3)	C6—C5—H5	119.8
C6—C1—Br	118.9 (3)	C4—C5—H5	119.8
C2—C1—Br	119.6 (3)	C3—C2—C1	118.5 (3)
C3—C4—C5	119.4 (3)	C3—C2—H2	120.7
C3—C4—C7	123.3 (3)	C1—C2—H2	120.7
C5—C4—C7	117.3 (3)	C2—C3—C4	121.0 (3)
C9—C8—C13	122.0 (3)	C2—C3—H3	119.5
C9—C8—O2	116.4 (3)	C4—C3—H3	119.5
C13—C8—O2	121.5 (3)	C5—C6—C1	119.0 (3)
C10—C11—C12	121.8 (3)	C5—C6—H6	120.5
C10—C11—N1	119.8 (3)	C1—C6—H6	120.5
C12—C11—N1	118.4 (4)	C8—C9—C10	118.9 (3)
C11—C10—C9	119.1 (3)	C8—C9—H9	120.5
C11—C10—H10	120.4	C10—C9—H9	120.5
C9—C10—H10	120.4	C12—C13—C8	118.9 (3)
O4—N1—O3	123.6 (4)	C12—C13—H13	120.5
O4—N1—C11	118.9 (4)	C8—C13—H13	120.5
O3—N1—C11	117.5 (4)	C13—C12—C11	119.2 (4)
O1—C7—O2	122.4 (3)	C13—C12—H12	120.4
O1—C7—C4	126.2 (3)	C11—C12—H12	120.4
O2—C7—C4	111.4 (3)		
			
C7—O2—C8—C9	123.0 (3)	C6—C1—C2—C3	1.3 (5)
C7—O2—C8—C13	−60.4 (4)	Br—C1—C2—C3	179.9 (3)
C12—C11—C10—C9	−1.7 (5)	C1—C2—C3—C4	−0.3 (5)
N1—C11—C10—C9	177.2 (3)	C5—C4—C3—C2	−1.2 (5)
C10—C11—N1—O4	−179.4 (4)	C7—C4—C3—C2	−179.6 (3)
C12—C11—N1—O4	−0.4 (6)	C4—C5—C6—C1	−0.7 (6)
C10—C11—N1—O3	0.0 (5)	C2—C1—C6—C5	−0.9 (6)
C12—C11—N1—O3	178.9 (4)	Br—C1—C6—C5	−179.5 (3)
C8—O2—C7—O1	0.6 (5)	C13—C8—C9—C10	1.5 (5)
C8—O2—C7—C4	−178.4 (3)	O2—C8—C9—C10	178.1 (3)
C3—C4—C7—O1	173.0 (4)	C11—C10—C9—C8	0.4 (5)
C5—C4—C7—O1	−5.4 (5)	C9—C8—C13—C12	−2.0 (6)
C3—C4—C7—O2	−8.0 (5)	O2—C8—C13—C12	−178.5 (3)
C5—C4—C7—O2	173.5 (3)	C8—C13—C12—C11	0.7 (6)
C3—C4—C5—C6	1.7 (6)	C10—C11—C12—C13	1.2 (6)
C7—C4—C5—C6	−179.8 (4)	N1—C11—C12—C13	−177.7 (3)

### Hydrogen-bond geometry (Å, °)

**Table:**

*D*—H···*A*	*D*—H	H···*A*	*D*···*A*	*D*—H···*A*
C10—H10···O4^i^	0.93	2.69	3.543 (6)	153.
C3—H3···O3^ii^	0.93	2.60	3.335 (5)	136.
C13—H13···O3^iii^	0.93	2.67	3.460 (5)	143.
C12—H12···O1^iv^	0.93	2.50	3.237 (5)	137.

Symmetry codes: (i) −*x*+1, *y*−1/2, −*z*+1/2; (ii) −*x*+1, −*y*, −*z*+1; (iii) *x*, −*y*+1/2, *z*+1/2; (iv) −*x*+1, −*y*+1, −*z*+1.
